# Characterization of Perinatal Stem Cell Spheroids for the Development of Cell Therapy Strategy

**DOI:** 10.3390/bioengineering10020189

**Published:** 2023-02-02

**Authors:** Francesca Paris, Pasquale Marrazzo, Valeria Pizzuti, Cosetta Marchionni, Maura Rossi, Martina Michelotti, Biljana Petrovic, Elisabetta Ciani, Giuliana Simonazzi, Andrea Pession, Laura Bonsi, Francesco Alviano

**Affiliations:** 1 Unit of Histology, Embryology and Applied Biology, Department of Medical and Surgical Sciences, University of Bologna, 40126 Bologna, Italy; 2 Department of Medical and Surgical Sciences, University of Bologna, 40138 Bologna, Italy; 3 Center for Applied Biomedical Research (CRBA), University of Bologna, 40138 Bologna, Italy; 4 Department of Biomedical and Neuromotor Science, University of Bologna, 40126 Bologna, Italy; 5 Obstetrics Unit, Department of Obstetrics and Gynecology, IRCCS Azienda Ospedaliero-Universitaria Sant’Orsola, 40138 Bologna, Italy; 6 Pediatric Unit, IRCCS Azienda Ospedaliero-Universitaria di Bologna, 40138 Bologna, Italy

**Keywords:** type 1 diabetes, amniotic membrane, umbilical cord, perinatal cells, placenta stem cells, amniotic epithelial cells, Wharton’s jelly, cell therapy, regenerative medicine, 3D culture, co-culture spheroids

## Abstract

Type 1 diabetes mellitus (T1DM) is a complex metabolic disease characterized by a massive loss of insulin-producing cells due to an autoimmune reaction. Currently, daily subcutaneous administration of exogenous insulin is the only effective treatment. Therefore, in recent years considerable interest has been given to stem cell therapy and in particular to the use of three-dimensional (3D) cell cultures to better reproduce in vivo conditions. The goal of this study is to provide a reliable cellular model that could be investigated for regenerative medicine applications for the replacement of insulin-producing cells in T1DM. To pursue this aim we create a co-culture spheroid of amniotic epithelial cells (AECs) and Wharton’s jelly mesenchymal stromal cells (WJ-MSCs) in a one-to-one ratio. The resulting co-culture spheroids were analyzed for viability, extracellular matrix production, and hypoxic state in both early- and long-term cultures. Our results suggest that co-culture spheroids are stable in long-term culture and are still viable with a consistent extracellular matrix production evaluated with immunofluorescence staining. These findings suggest that this co-culture may potentially be differentiated into endo-pancreatic cells for regenerative medicine applications in T1DM.

## 1. Introduction

Type one diabetes mellitus (T1DM) is a complex autoimmune disorder characterized by the immune-mediated destruction of insulin-producing beta cells. This condition leads not only to increased blood glucose levels but also to alterations in the micro and macro blood circulation, which implies an increased risk to develop nephropathy, retinopathy, and neuropathy [1]. Currently, T1DM therapy consists chiefly of daily administration of exogenous insulin via multiple subcutaneous injections or through insulin infusion. Unfortunately, insulin mono-therapy presents a considerable risk of hypoglycemia and strict control of glucose levels is mandatory. For these reasons, new frontiers of therapies for T1DM are being studied in recent years to treat the disease but also hopefully to reverse it in the future. These therapies include immunotherapies that target T cells as well as cell therapy [2]. The differentiation of various stem and progenitor cell populations into functional islet cells has provided new hope for the field [3]. Indeed, the use of stem cells could be a valid approach to making progress in the understanding of biological systems and, in the future, to the development of new cellular therapy. Among the various sources of cells used in cell therapy, particular interest has been given in the past 20 years to perinatal cells and their potential application in regenerative medicine. Perinatal cells are isolated from term placentas and fetal annexes. Since perinatal tissues are discarded immediately after birth the resulting cells are free of ethical implications and present several advantages, including their easy collection and the high yield in terms of cell number obtained after isolation [4,5,6].

In our study, we focus on two different perinatal cells: amniotic epithelial cells (AECs), isolated from the inner part of the amniotic membrane, and Wharton’s jelly mesenchymal stromal cells (WJ-MSCs), isolated from the umbilical cord. Due to their early development, perinatal cells retain characteristics found in pluripotent stem cell populations and have been successfully induced to differentiate towards all three germ layers under specific culture conditions [7]. Importantly, AECs and WJ-MSCs have been successfully differentiated into the pancreatic–endodermic lineage [4,8,9,10]. Moreover, cell populations deriving from the placenta are physiologically involved in the achievement of feto-maternal tolerance, whose role is to avoid the immune-mediated rejection of the embryo during pregnancy. The immunomodulatory properties of perinatal cells are a fundamental aspect for regenerative medicine applications and for the treatment of auto-immune diseases, including T1DM [11].

Thanks to the advancements in matrices and scaffold engineering, studies on three-dimensional (3D) cultures have increased in recent years. Spheroid formation is usually achieved through the use of these techniques: (a) forced floating, (b) hanging drop, (c) spinning flask, (d) rotating vessel, (e) electrical-force assisted, (f) magnetic-force assisted, and (g) matrix-based [12]. Three-dimensional models mimic the in vivo cell environment better than a classical two-dimension (2D) cell culture, improving cell viability, morphology, proliferation, response to stimuli, and differentiation. Moreover, 3D cultures have greater stability and longer lifespans than cell cultures in 2D.

Among 3D models, spheroids are dense cellular structures that are promising to increase the therapeutic potential of cell-based therapies. Specifically, spheroids retain their extracellular matrix (ECM), which is fundamental to increasing cell survival in harsh conditions and up-regulating trophic factor secretion. Thanks to the presence of ECM and cell–cell interactions, spheroids better mimic the micro-environment found in native tissue [3,12]. Moreover, spheroids can be constituted of different types of cells giving rise to more physiological cell–cell interactions. The aim of our work is to create a 3D model consisting of a co-culture of AECs and WJ-MSCs. In this way, it is possible to combine the ability of AECs to differentiate into insulin-producing cells, already demonstrated by our laboratory [4], with the scaffolding support and ECM secretion exerted by WJ-MSCs, which are crucial for the stability of a 3D model [13]. A co-culture of epithelial cells and mesenchymal cells could be a valid model to mimic the pancreatic micro-environment in which a tight correlation between glandular cells and stromal cells is present. One more important aspect is that both AECs and WJ-MSCs have immunomodulatory activity, specifically by stimulating the tolerogenic cell component. In the circumstance of T1DM, the immunomodulatory effect could be a valid approach to preserve beta cells from the hyperactivation of the immune system [14].

## 2. Materials and Methods

### 2.1. Ethics Statement

This study was approved by the Local Ethical Committee (IRCCS St. Orsola-Malpighi University Hospital Ethical Committee, protocol n° 2481/2017, ref n° 68/2017/U/Tess). Placentas were obtained from healthy donor mothers undergoing elective caesarean section at term (37–40 weeks) after written informed consent. Perinatal tissues were maintained under sterile conditions until specific cell isolation was performed.

### 2.2. Isolation of Human Amniotic Epithelial Cells (AECs)

Fetal membranes were washed with ice-cold phosphate-buffered saline (PBS, Corning, NY, USA) with 1% penicillin–streptomycin solution (10,000 U/mL penicillin, 10,000 U/mL streptomycin, Corning, Steuben County, NY, USA). The amniotic membrane was mechanically peeled off the underlying chorion layer to remove any blood clots. Then the tissues were incubated for 10 min at room temperature with PBS/ethylenediaminetetraacetic acid (EDTA) 0.5 mM. The amniotic membrane was then minced into small pieces (4 cm^2^ approximately) and digested twice for 30 min at 37 °C using trypsin-EDTA 0.25% (Corning, Steuben County, NY, USA) with gentle shaking. For both digestion steps, trypsin was inactivated with fetal bovine serum (FBS, Gibco, Life Technologies, Carlsbad, CA, USA) and the cell suspension was centrifuged for 10 min at 390× *g*. The cell pellet was resuspended in basal culture medium, Dulbecco’s Modified Eagle’s Medium–high glucose (DMEM H., Corning, Steuben County, NY, USA) containing 10% FBS, 1% penicillin-streptomycin solution, and 10 ng/mL of epithelial growth factor (EGF, Sigma-Aldrich, St. Louis, MO, USA). The single-cell suspension was counted and tested for viability using erythrosin B (Sigma-Aldrich, St. Louis, MO, USA). Only samples with >90% viability were used for further assays.

### 2.3. Isolation of Wharton’s Jelly Mesenchymal Stromal Cells (WJ-MSCs)

First, the umbilical cord (UC) was removed from the placenta. UC was minced into pieces of approximately 2–3 cm lengths and rinsed 3–4 times with phosphate-buffered saline (PBS, Corning, NY, USA) containing 1% penicillin–streptomycin (10,000 U/mL penicillin, 10,000 U/mL streptomycin, Corning, Steuben County, NY, USA). After the removal of two arteries and a vein, Wharton’s jelly (WJ) was chopped into pieces of 3–5 mm with a scalpel. These small pieces were transferred onto the culture dishes and covered with a drop of Dulbecco’s Modified Eagle’s Medium (DMEM) with 10% FBS (fetal bovine serum) to prevent the pieces of WJ from drying out. The dishes were placed in the incubator with 5% CO_2_ for at least two hours and then 10 mL of medium was added to the dishes. The culture dishes were maintained and left untouched for 5 days in the incubator. On day 5 of culture, 3 mL of media was added. After approximately 7–14 days, WJ-MSCs were isolated. The explanted tissues were gently removed and the isolated cells were detached by the surface using Accutase^®^ (P10-21500, Pan Biotech, Bayern, Germany) and centrifuged at 300× *g* for 5 min. After centrifugation, cells were recovered and the single-cell suspension was counted and tested for viability using erythrosin B (Sigma-Aldrich, St. Louis, MO, USA).

### 2.4. Immunophenotype of AECs and WJ-MSCs

Immunophenotypic characterization of isolated AECs and WJ-MSCs was performed using flow cytometry. Cells were fixed for 10 min at room temperature using Intraprep Kit (Beckman–Coulter Inc., Brea, CA, USA) and washed twice with PBS. Cells were incubated for 30 min at 4 °C with conjugated primary antibodies (1 μg/mL) specific for epithelial (anti-pan Cytokeratin (Pan-Ck)-PE, Santa Cruz Biotechnology, Santa Cruz, CA, USA), mesenchymal (anti-CD44-PE, anti-CD73-PECY7, anti-CD90-FITC, anti-CD105-PECY7, Beckman-Coulter Inc., Brea, CA, USA), hematopoietic (anti-CD14-PE, anti-CD34-PerCP, anti-CD45-FITC, Beckman-Coulter Inc., Brea, CA, USA), and stem cell (anti-state-specific embryonic antigen-4 (SSEA4) APC, Miltenyi Biotech, Germany) markers. For the analysis of Pan-Ck, we used the Pan-Ck Type I/II Antibody Cocktail (MA5-13156, Thermo Scientific, Waltham, MA, USA) and anti-mouse–Alexa Fluor 488 (A11001, Thermo Scientific, Waltham, MA, USA) as a secondary antibody. After incubation, cells were washed with PBS and analyzed using the FACS Navio FC (Beckman-Coulter Inc., Brea, CA, USA) cytometer and the Kaluza FC C 1.2 Analysis software (Beckman-Coulter Inc., Brea, CA, USA).

### 2.5. 3D Culture, Perinatal Spheroid Generation

Spheroids were generated starting from isolated WJ-MSCs seeded as mono-culture or by combining WJ-MSCs with AECs using 96-well round-bottom cell-repellant plates (Greiner Bio-One, Kremsmünster, Austria). Briefly, 200 μL of DMEM with 10% FBS containing a total of 5000 cells/well were seeded in each well in both mono- and co-culture spheroids. In the co-culture setting, 2500 cells/well of each cellular subtype and EGF (10 ng/mL) were added. Immediately after seeding, the plates were centrifuged at 50× *g* for 3 min and placed in an incubator at 37 °C with 5% CO_2_. The culture media was changed every day thereafter.

### 2.6. Spheroid Formation

Co-culture spheroids were generated starting from isolated WJ-MSCs stained using Vybrant™ DiD Cell-Labeling Solutions (Molecular Probes, Eugene, OR, USA) and AECs stained using Vybrant™ DiO Cell-Labeling Solutions (Molecular Probes, Eugene, OR, USA). Biefly, 1 × 10^6^ cells/mL in serum-free DMEM were incubated for 20 min at 37 °C with the corresponding labelling solution (5 μL/mL). Cells were washed twice in PBS and seeded in 96-well round-bottom cell-repellant plates. Immediately after seeding, the plates were centrifuged at 50× *g* for 3 min and placed in the IncuCyte^®^ S3 live imaging system (Sartorius) for 96 h at 37 °C in 5% CO_2_. Bright-field and immunofluorescence images were acquired using 10× magnification every 3 h from the seeding to the complete spheroid formation after 96 h.

### 2.7. Prestoblue^®^ Assay for Spheroid Viability

To determine the presence of metabolically active cells in our spheroids the resazurin reduction assay was performed [15]. The assay is based on the principle that resorufin, which is the reduced form of resazurin, can be measured using a fluorescent method, thus indirectly determining the viability rate of incubated spheroids. Following 4 and 14 days of 3D culture, the spheroids were transferred to a non-ULA 96 well plate and incubated for 24 h to allow the spheroids to adhere. After 24 h 100 μL of 10% Prestoblue^®^ stock solution was added and incubated for a maximum of 24 h at 37 °C under 5% CO_2_ humidified conditions. The reduction of the reagent was measured at 595 nm at 2, 4, 6, 8, and 24 h using a multiplate reader (Victor II—Perkin Elmer, Waltham, MA, USA). 

### 2.8. Live and Dead Staining

Cell viability within the spheroids was tested using the Live/Dead Cell Assay Kit (Life Technologies). This assay was used to visually determine the group of cells within spheroids maintaining viability after clusterization indispensable for spheroid formation. Spheroids of mono- and co-culture at 4 and 14 days were incubated with a solution containing 1 μM calcein AM and 2 μM ethidium homodimer-1 at 37 °C for 45 min. After washing with PBS, spheroids were imaged with laser excitation of the sample at 488 nm and 561 nm using a Nikon Inverted Microscope (Nikon Instruments, Tokyo, Japan), and images were acquired with a Digital Sight camera DS-03 using the imaging software NIS-Elements 4.1 (Nikon Corporation, Tokyo, Japan).

### 2.9. Slide Preparation and Histology

The spheroids were washed in PBS and transferred to 1.5 mL Eppendorf tubes to encapsulate them with Epredia™ HistoGel™ Specimen Processing Gel (Fisher-Scientific, Loughborough, UK). Each gel specimen was moved to a cassette and loaded inside a tissue processor (Histo-line laboratories, Pantigliate, Italy). The samples were embedded in paraffin blocks using an automated inclusor (Medite Medical, Burgdorf, Germany). Microtome (Microme, Bio-Optica, Milano, Italy) sections of 10 µm were placed on Super Frost glass slides (DiaPath, Menzel, Martinengo, Italy) and allowed to dry. The sections were deparaffinized with 2 changes of xylene for 5 min and rehydrated with 2 changes of 100% ethanol, followed by washes in 90% ethanol and 70% ethanol for 3 min. Finally, the slides were rinsed in distilled water.

### 2.10. Hematoxylin and Eosin Staining

The slides were deparaffinized, rehydrated, and stained with hematoxylin and eosin. The sections were stained with Gill 2 hematoxylin (Bio-Optica) for 5 min, washed in tap water for 3 min, rinsed with distilled water, and subsequently stained with 1% eosin G (Bio-Optica) for 10 min, dehydrated in ethanol of ascending concentration (70, 90, and 100%), clarified in pure xylene, and mounted with Bio Mount balm (Bio-Optica). Stained sections were observed using a Leica DM 750 equipped with a Leica ICC50 HD digital camera.

### 2.11. Biophysical Properties Analysis of Spheroids (W8 Analysis)

The W8 instrument (CellDynamics SRL) is able to accurately measure the size, weight, and density of the spheroids. Single spheroids free-fall into a vertical flow channel dedicated to analyzing their terminal velocity. The physical approach of this analysis and the mathematical equations used for the calculation is extensively described by Cristaldi et al. [16]. Before the measurement, the spheroids were fixed with paraformaldehyde (PFA) 4% for 72 h at 4 °C, resuspended in 4 mL of phosphate-buffered saline (PBS, Corning, NY, USA), transferred in a centrifuge conical tube, and then analyzed. A minimum of 10 spheroids was analyzed for both mono- and co-culture at different time points. 

### 2.12. Immunofluorescence Analysis

Antigen retrieval was performed by incubating slides of sectioned spheroids in sodium citrate pH 6 at 95 °C for 30 min. Sections were washed with PBS and permeabilized by adding PBS 0.3% Triton (Triton X-100, Sigma-Aldrich, Co., St. Louis, MO, USA) for 10 min. Slides were incubated for 30 min with a blocking solution containing PBS 4% bovine serum albumin (BSA, Sigma-Aldrich, St. Louis, MO, USA) and 0.3% Triton, then incubated overnight at 4 °C with the primary antibodies anti-vimentin (1:200, #MA5-14564, Thermo Fisher Scientific, Waltham, MA, USA), anti-pan cytokeratin (1:200, #MA5-13156, Thermo Fisher Scientific, Waltham, MA, USA), anti-laminin (1:100, #L9393, Sigma-Aldrich, Co., St. Louis, MO, USA), anti-fibronectin (1:200, #F3648, Sigma-Aldrich, Co., St. Louis, MO, USA), anti-cadherin (1:200, #71-7100, Thermo Fisher Scientific, Waltham, MA, USA), and anti-collagen I (1:200, #BK72026S, Cell Signaling Technology, Danvers MA, USA) diluted in blocking solution. Secondary anti-mouse Alexa Fluor 488 (1:500, #A11001, Thermo Scientific, Waltham, MA, USA) and Alexa Fluor 594 (1:500, #A32754, Thermo Fisher Scientific, Waltham, MA, USA) were used for 1 h incubation at room temperature. After three washes with PBS, coverslips were mounted using the Prolong Gold Antifade Mountant with DAPI (Thermo Fisher Scientific, Monza, Italy). Stained cells were observed using a Nikon Inverted Microscope (Nikon Instruments, Tokyo, Japan), and images were acquired with a Digital Sight camera DS-03 using the imaging software NIS-Elements 4.1 (Nikon Corporation, Tokyo, Japan).

### 2.13. Hypoxia Probe

The hypoxia level of whole-mounted spheroids was evaluated using the hypoxia LOX-1 probe (Organogenix- MBL) according to the manufacturer’s instructions. The probe was added to the culture medium at a final concentration of 2 μM 24 h before detection. Images visualizing the hypoxic area in mono- and co-culture spheroids were obtained using a Nikon Inverted Microscope (Nikon Instruments, Tokyo, Japan), and images were acquired with a Digital Sight camera DS-03 using the imaging software NIS-Elements 4.1(Nikon Corporation, Tokyo, Japan). The percentage of the hypoxic area within a total spheroid area was determined.

### 2.14. Statistical Analysis

All the experiments, except the one for which details were already described, were performed on at least three human samples in technical triplicate. Data are presented as mean ± standard deviation (SD) and were analyzed with two-way ANOVA or *t*-test using Graph Pad Prism 9.0 software (San Diego, CA, USA). The significance threshold was set at *p* < 0.05.

## 3. Results

### 3.1. Immunophenotypic Characterization of Isolated AECs and WJ-MSCs

**Figure 1:**
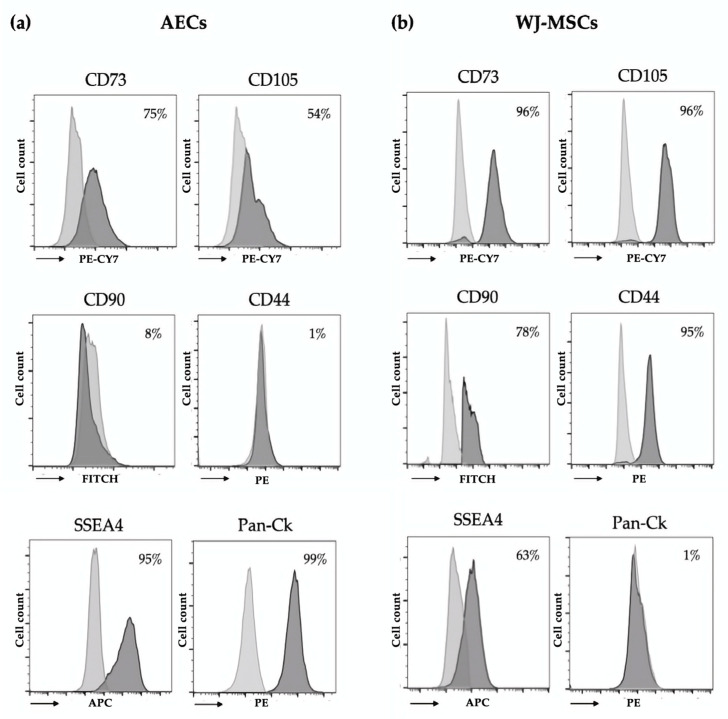
Immunophenotype of isolated AECs and WJ-MSCs. Data are representative of three independent experiments obtained from three human samples. Grey and black histograms represent the unstained controls and the specific cell markers, respectively. (**a**) Flow cytometry analysis of AECs: mesenchymal (CD44, CD73, CD90, CD105), stem cell (state-specific embryonic antigen-4, SSEA4), and epithelial (Pan-Cytokeratin, Pan-Ck) markers. (**b**) Flow cytometry analysis of WJ-MSCs: mesenchymal (CD44, CD73, CD90, CD105), epithelial (Pan-Ck), and stem cell (SSEA4) markers.

The immuno-phenotypic profile of isolated AECs and WJ-MSCs was characterized using a flow cytometry approach. The epithelial, mesenchymal, and hematopoietic markers were evaluated. As shown in Figure 1a, AECs expressed high levels of Pan-Ck, confirming their epithelial origin. AECs were also positive for the surface markers CD73 and CD105, while negative for CD44 and CD90, in agreement with recent studies [17]. These results are in line with previous studies [4,15]. Concerning the mesenchymal counterpart, WJ-MSCs were positive for all mesenchymal surface markers (CD73, CD90, CD105, and CD44) and negative for the epithelial marker (Pan-Ck), as shown in Figure 1b. Moreover, both AECs and WJ-MSCs were positive for the stem cell marker SSEA4. Hematopoietic markers such as CD14, CD34, and CD45 were not expressed in both AECs (Figure S1a) and WJ-MSCs (Figure S1b).

### 3.2. Spheroid Formation

**Figure 2:**
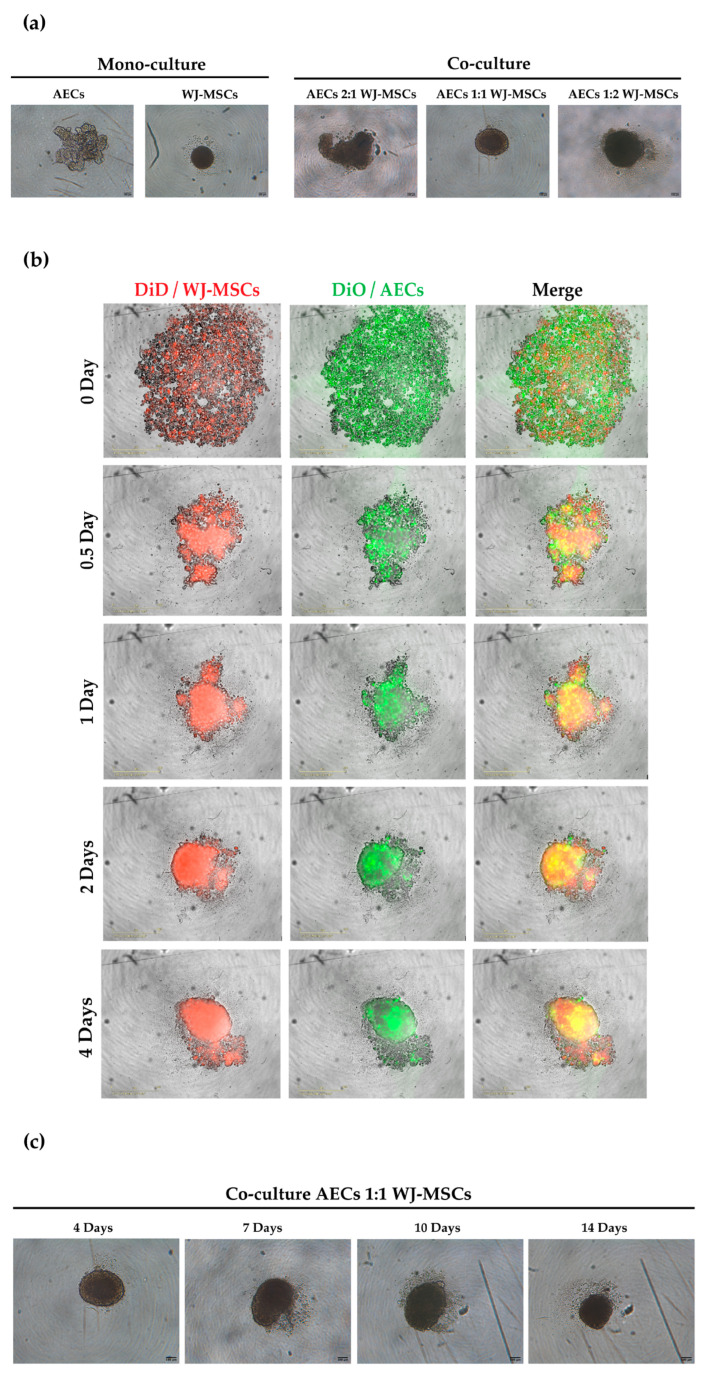
Representative images showing mono-culture spheroids of AECs and WJ-MSCs and co-culture spheroid formation at different ratios. (**a**) Mono-culture spheroid of AECs (5000 cells/spheroid) and WJ-MSCs (5000 cells/spheroid) and co-culture of AECs and WJ-MSCs with three different ratios of AECs:WJ-MSCs—2:1, 1:1, 1:2 (total 5000 cells/spheroid)—after 4 days of culture. Images were acquired using a Leica Labovert FS inverted microscope equipped with a Leica MC170 HD digital camera. Scale bars = 100 µm, magnification = 4×, N = 3 independent experiments. (**b**) Representative images of 1:1 co-culture spheroid formation at 0, 0.5, 1, 2, and 4 days using the IncuCyte^®^ S3 live imaging system. WJ-MSCs were stained with DiD cell-labeling solution (red) and AECs were stained with DiO cell-labeling solution (green). Scale bars = 400 µm. Magnification = 10× (**c**) Long-term culture of 1:1 ratio spheroids. Images were acquired using a Leica Labovert FS inverted microscope equipped with a Leica MC170 HD digital camera. Scale bars = 100 µm, magnification = 4×, N = 3 independent experiments.

To enhance cell–cell interactions, cells were seeded in cell-repellent plates, resulting in the formation of three-dimensional structures (spheroids, Figure 2a). In our study, two different cell types were tested for spheroid formation: AECs and WJ-MSCs. After seeding, the cells started to self-assemble in the well. After 4 days of incubation, WJ-MSC mono-cultures formed aggregates that were stable and easily handled, generating compact, rigid, spherically-shaped spheroids. Differently, AECs do not self-assemble in spheroids, but created a loose, compact sheet of cells that is easily dissociated. To set up a co-culture of AECs and WJ-MSCs that could display features of both cell types we tested three different ratios of AECs to WJ-MSCs: 2:1, 1:1, and 1:2. Spheroids with a 2:1 ratio were less compact and did not show spherical shapes. On the contrary, spheroids with a ratio of 1:2 recapitulated the appearance of WJ-MSC mono-culture with compact, rigid, spherically-shaped spheroids. To maintain the highest number of AECs, we considered as appropriate the 1:1 ratio. 

In order to visualize cell aggregation at an early time point we followed the cell culture from seeding to complete spheroid formation (4 days) using the IncuCyte^®^ S3 live imaging system. As shown in Figure 2b, WJ-MSCs rapidly condensed in a cell mass, while AECs were distributed on the surface of the spheroid, increasing their compactness over time. 

Since the majority of the differentiation protocols in endo-pancreatic linages consist of 14 days of culture [4] we tested the stability of long-term culture. As shown in the representative images in Figure 2c, the co-culture spheroid maintained its shape and did not disaggregate during long-term culture.

### 3.3. Evaluation of Spheroid Viability

**Figure 3:**
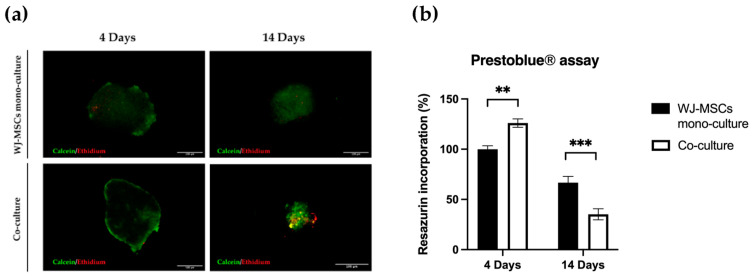
Viability of mono-culture WJ-MSC (black) spheroids and co-culture (white) spheroids. (**a**) Metabolic activity was measured with a Prestoblue^®^ assay at 4 and 14 days in mono- and co-culture spheroids. N = 4 independent experiments. ** *p* < 0.01; *** *p* < 0.001; (**b**) Cell viability assay of spheroids in mono- and co-culture at 4 and 14 days, evaluated by staining with ethidium homodimer-1 and calcein-AM. Images are representative of three independent experiments. Green, viable cells; red, non-viable cells. Cells were observed using a Nikon Inverted Microscope (Nikon Instruments, Tokyo, Japan), and images were acquired with a Digital Sight camera DS-03 using the imaging software NIS-Elements 4.1 (Nikon Corporation, Tokyo, Japan). Scale bar = 100 µm. Magnification = 10×.

The viability of cells within the spheroids was evaluated using a Live/Dead Cell Assay Kit. The ethidium homodimer-1 staining indicates a compromised cell membrane (red fluorescence), and calcein fluorescence highlights viable cells (green fluorescence) (Figure 3a). The WJ-MSC mono-culture appeared viable at 4 and 14 days. Co-culture spheroids appeared viable at 4 days, but the cell viability in the inner part was considerably compromised at 14 days. Metabolic activity of mono- and co-culture spheroids was also evaluated at 4 and 14 days using a Prestoblue^®^ assay, as shown in Figure 3b. At 4 days, co-culture spheroids expressed higher metabolic activity than the WJ-MSC mono-culture, while at 14 days a considerable decrease in the metabolic activity of both mono- and co-culture was detected. In accordance with the Live/Dead Cell Assay Kit, we assessed that WJ-MSC spheroids are viable and more metabolically active compared to co-culture.

### 3.4. Histological Analysis

**Figure 4:**
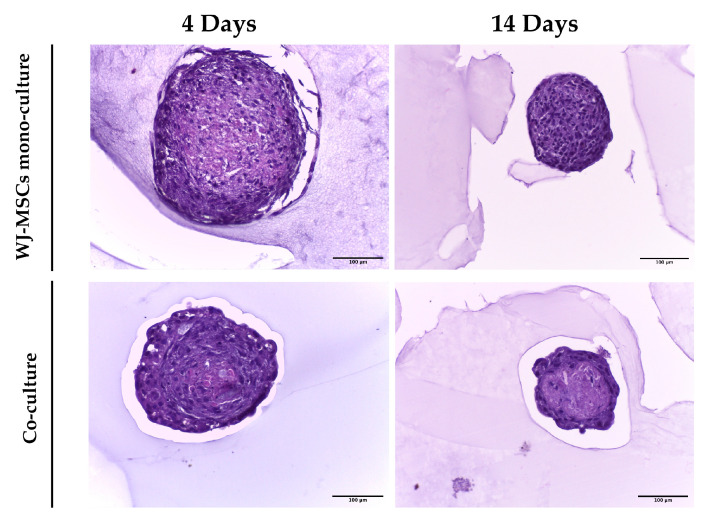
Hematoxylin and eosin staining on WJ-MSC mono-culture and co-culture spheroid sections after 4 and 14 days of culture. Spheroids were fixed in 4% PFA, then encapsulated with Epredia™ HistoGel™ Specimen Processing Gel (Fisher-Scientific, UK), sectioned, and then stained with hematoxylin and eosin. Images were acquired using a Leica DM 750 equipped with a Leica ICC50 HD digital camera. Scale bars = 100 µm. Magnification = 20×.

Hematoxylin and eosin staining was performed on spheroid sections at the early and late time points for both WJ-MSC mono-culture and co-culture. In Figure 4, WJ-MSC spheroids present a homogeneous structure and defined edges in both early- and late- time point spheroids. In contrast, at an early time point, co-culture spheroids have a heterogeneous structure and irregular edges given by the presence of the two cell populations. At 14 days the co-culture displays an external ring of cells and a core characterized by a reduced number of nuclei and extensive ECM production.

### 3.5. Characterization of the Physical Properties of Mono- and Co-Culture Spheroids

**Figure 5:**
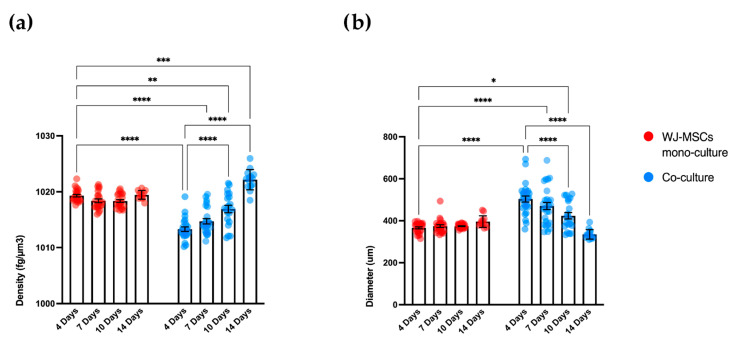
Measurement of mass density and diameter of mono-culture WJ-MSCs spheroids (red dots) and co-culture (WJ-MSCs + AECs) spheroids (blue dots). (**a**) Analysis of spheroid density (fg/µm^3^) at four time points: 4, 7, 10, and 14 days for both mono- and co-culture spheroids. The density of WJ-MSC spheroids remains stable over time while co-culture spheroids increase their density. (**b**) Analysis of spheroid diameter (µm) at four time points: 4, 7, 10, and 14 days for both mono- and co-culture spheroids. The diameter of WJ-MSC spheroids remains stable over time similarly to the density while co-culture diameter decreases with the increase in the density. N = 4 independent experiments. * *p* < 0.05; ** *p* < 0.01; *** *p* < 0.001; **** *p* < 0.0001.

We analyzed mono-cultures of WJ-MSCs and co-cultures of WJ-MSCs and AEC spheroids obtained as described above. At least ten single spheroids for each condition were analyzed at four time points: 4, 7, 10, and 14 days of 3D culture. For each spheroid, the diameter and the mass density were analyzed. Experiments were performed on a homogeneous population of spheroids in terms of cell number (5000 cells/spheroid) to evaluate the contribution of each cell population in terms of density and diameter. Mono- and co-culture spheroid samples were fixed with 4% PFA and analyzed with the flow-based system. The diameter of WJ-MSC spheroids (μm), calculated automatically from the images acquired during the analysis, was stable during the four time points, as was the mass density (Figure 5). The average diameter of co-culture spheroids was progressively reduced over time, as shown in Figure 5a. The reduction in the diameter of the co-cultures was associated with an increase in mass density (Figure 5b), suggesting not a loss in whole cell number but rather an increase in compactness and matrix production. 

### 3.6. Expression of Pan-Cadherins in Mono- and Co-Culture Spheroids

**Figure 6:**
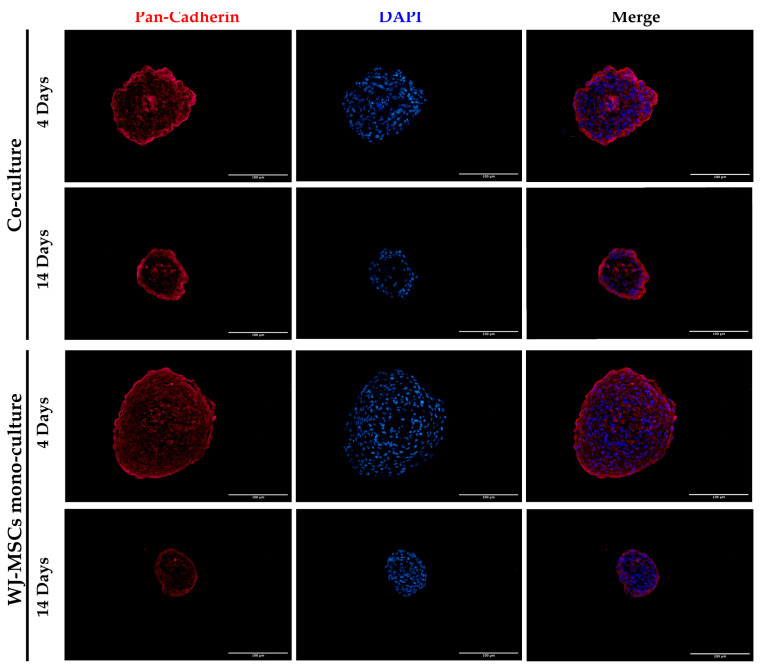
Representative images of WJ-MSC mono-culture and co-culture spheroids at early (4 days) and late (14 days) time points stained with Pan-Cadherin (red) and DAPI for nuclei staining (blue). Cells were observed using a Nikon Inverted Microscope (Nikon Instruments, Tokyo, Japan), and images were acquired with a Digital Sight camera DS-03 using the imaging software NIS-Elements 4.1 (Nikon Corporation, Tokyo, Japan). Scale bar = 100 µm. Magnification = 20×.

We investigated the presence of cadherin molecules in mono- and co-culture spheroids to evaluate cell–cell interactions. Cadherins are fundamental players in spheroid formation as they create homophilic cadherin–cadherin binding that contributes to spheroid compaction [15,18]. Cadherin’s type and concentration are highly variable between different cell types; thus, we evaluated the presence of all cadherins with pan-cadherin antibody staining on the spheroid sections [15]. In Figure 6, cadherins accumulated after the first time point and their staining were maintained in long-term cultures, indicating a strong cell–cell interaction, in particular on the external surface of the spheroids. 

### 3.7. Pan-Cytokeratins and Vimentin Expression in Mono- and Co-Cultures

**Figure 7:**
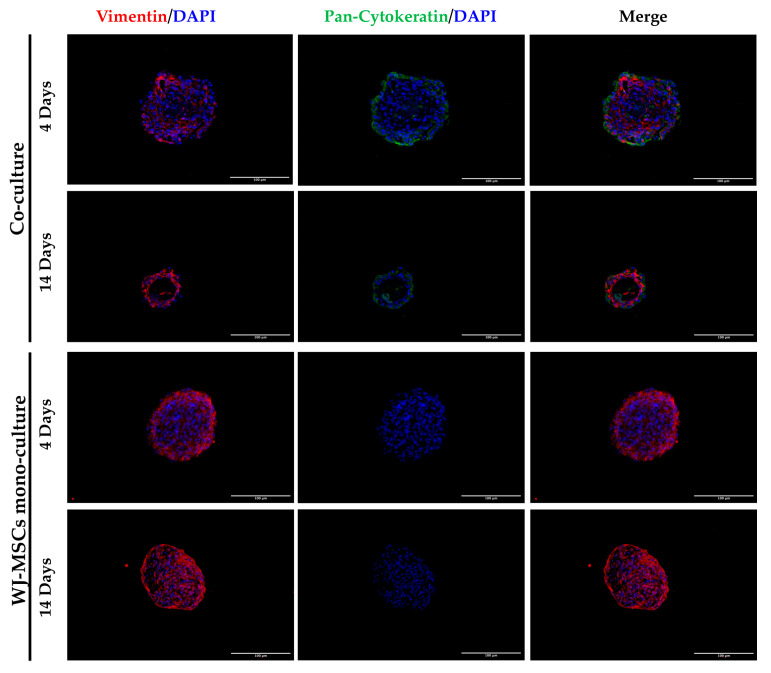
Representative images of WJ-MSC mono-culture and co-culture spheroids at early (4 days) and late (14 days) time points stained with Pan-Ck (green), vimentin (red), and DAPI for nuclei staining (blue). Cells were observed using a Nikon Inverted Microscope (Nikon Instruments, Tokyo, Japan), and images were acquired with a Digital Sight camera DS-03 using the imaging software NIS-Elements 4.1 (Nikon Corporation, Tokyo, Japan). Scale bar = 100 µm.

As the formation of the spheroids occurred through a self-organization process, we wanted to further characterize the structure of our 3D model co-culture to inspect the arrangement of the two cellular components within the spheroid and evaluate possible alterations during the long-term culture. Thanks to the immunofluorescence staining of the epithelial cell marker Pan-Ck (Figure 7), it was possible to appreciate that AECs covered the entire spheroid’s surface, while only a few Pan-Ck positive cells were entrapped in the inner part of the 3D structure. On the contrary, no positivity for Pan-Ck staining was observed in WJ-MSC mono-cultures. WJ-MSCs were visualized as single-positive for vimentin expression in co-cultures. In co-culture spheroids, at both 4 and 14 days, AECs co-expressed cytokeratin and vimentin, as already reported [19]. At 14 days co-culture spheroids reduced their diameter and in the core of the spheroid there was a reduced number of nuclei. Even though the expression of Pan-Ck did not change, degeneration during long term-culture were observable in WJ-MSCs.

### 3.8. Extracellular Matrix Formation

**Figure 8:**
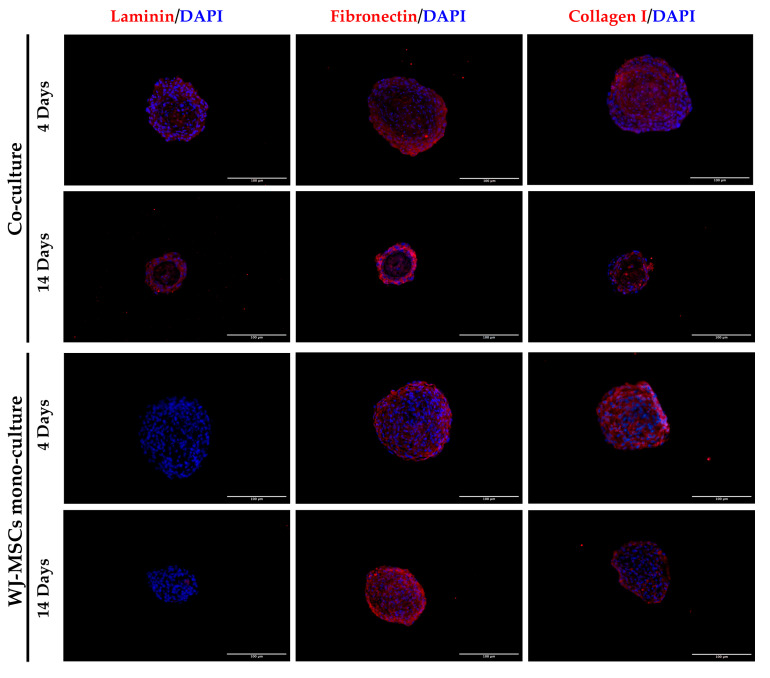
Representative images of WJ-MSC mono-culture and co-culture spheroids at early (4 days) and late (14 days) time points stained to evaluate the presence of ECM proteins with laminin, fibronectin, and collagen I (red) and DAPI for nuclei staining (blue). Cells were observed using a Nikon Inverted Microscope (Nikon Instruments, Tokyo, Japan), and images were acquired with a Digital Sight camera DS-03 using the imaging software NIS-Elements 4.1(Nikon Corporation, Tokyo, Japan). Scale bar = 100 µm. Magnification = 20×.

We evaluated the presence of ECM proteins in our spheroids. ECM is widely known to provide structural support in tissues and also plays important roles in cell behavior, including cell adhesion, migration, and compartmentalization [20]. In particular, the presence of three different components was assessed. Figure 8 shows laminin, fibronectin, and type I collagen immunofluorescence staining. As observed in Figure 8, laminin was not observed in WJ-MSC mono-cultures, but it was detected in co-cultures, particularly in correspondence with the epithelial cell layer. The production of laminin by epithelial cells in a 3D model of AECs has also been observed in our previous study [4] and recapitulated in vivo tissue organization. Type I collagen is the most prevalent ECM component providing structural support [21], while fibronectin is mainly responsible for cell adhesion and cellular migration [20,22]. In both mono- and co-culture, fibronectin and type I collagen were expressed even at early time points, establishing a 3D ECM network. The presence of an ECM network, which is usually inconsistent in 2D culture, represents an important structural support for the artificial reconstruction of pancreatic tissue [22]. 

### 3.9. Evaluation of Hypoxia 

**Figure 9:**
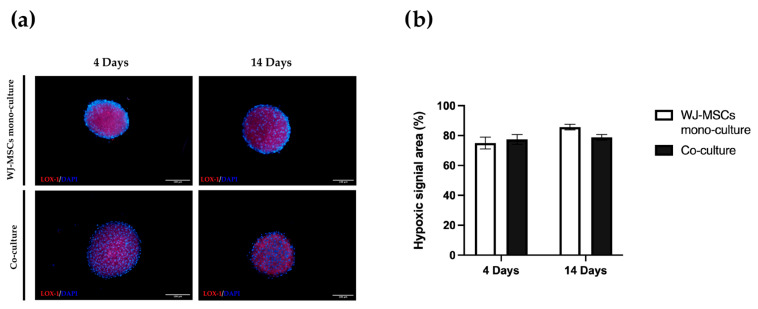
Hypoxia levels in mono-culture WJ-MSC spheroids and co-culture spheroids. (**a**) Images are representative of three independent experiments. Hypoxic signal in red (LOX-1); cell nuclei in blue (DAPI). Cells were observed using a Nikon Inverted Microscope (Nikon Instruments, Tokyo, Japan), and images were acquired with a Digital Sight camera DS-03 using the imaging software NIS-Elements 4.1 (Nikon Corporation, Tokyo, Japan). Scale bar = 100 µm. Magnification = 10×. (**b**) Hypoxia probe quantification was calculated as percentage of hypoxic area on the total spheroid area using ImageJ software v1.53k. WJ-MSC mono-culture (white); co-culture (black). N = 4 independent experiments.

To further characterize WJ-MSC mono-culture and co-culture spheroids we evaluated the hypoxic state at 4 and 14 days. A hypoxia LOX-1 probe (Figure 9) was used and its signal was acquired with the fluorescent microscope. Red fluorescence indicated a low oxygen level inside the multicellular spheroids. As shown in Figure 9a, a spread inner hypoxic signal was detected in a large area of both mono-culture and co-culture spheroids. The levels of hypoxia did not significantly change among spheroids after long-term culture (14 days) (Figure 9b).

## 4. Discussion

Currently, T1DM therapy is based almost exclusively on the control of insulin levels through the administration of exogenous insulin. Patients with diabetes have a high risk of incurring other pathologies, which can considerably shorten life expectancy [23,24]. Alternative solutions are highly demanded to find new treatments for diabetes. An ideal therapeutic approach may act on the reduction of both blood glucose levels and auto-immune response. In this context, cell therapy can be considered a suitable option.

Perinatal cells offer a wise solution as a candidate cell source for cell therapy against T1DM [2]. In particular, AECs and WJ-MSCs have immunomodulatory activity, inhibiting the activation of the immune system and stimulating the tolerogenic cell component. In the context of T1DM, the immunomodulatory properties exerted by perinatal cells could be a valid tool to preserve beta cells from the hyper-activation of the immune system [10]. Thus, our work aims to develop a 3D cellular model that is suitable for endo-pancreatic differentiation protocols as a possible step in the development of a cell therapy against T1DM. Three-dimensional models of WJ-MSC, such as spheroids, show an increased physiological relevance for future clinical applications based on their greater immunomodulatory and differentiating properties [25]. While the assembly of WJ-MSC spheroid mono-cultures has been reported and largely characterized, less information is available for AEC spheroids. Our group has already developed 3D cultures of AECs, which were shown to be able to secrete insulin and glucagon [4], thus representing a good cell source for endocrine pancreatic differentiation. However, the levels of insulin secreted were not high enough to be considered physiologically relevant and, as shown in our experiment, AECs alone have difficulty forming spheroids with regular edges and roundness (Figure 2). For this reason, establishing a new optimized 3D model is fundamental. To address this, we develop a 3D spheroid co-culture of WJ-MSCs and AECs. WJ-MSCs act as structural support for the development of more consistent spheroids. Moreover, the addition of a mesenchymal population can better mimic the structural and functional characteristics of native tissue micro-environments, such as pancreatic islets, where epithelial and stromal cells are closely connected. Furthermore, WJ-MSCs have several advantages in spheroid formation, including the secretion of factors that promote tight junctions, cell–cell adhesion, and extracellular matrix formation. Additionally, WJ-MSCs have been found to enhance the production of growth factors and cytokines, and have increased differentiation and stemness potential, which could increase insulin secretion [26,27]. 

As a first step in creating co-cultures, we combined AEC and WJ-MSC cultures in different ratios. By using a ratio enabling a double quantity of AECs in comparison to the WJ-MSCs, co-cultures did not efficiently form spheroid-like shapes. Among the tested ratios, the most suitable and consistent cell aggregation was found in the one-to-one ratio. Co-culture in a one-to-one ratio appeared to store all cell types and have a consistent and reproducible shape and dimensions among donors and repeated experiments. Moreover, the diameter of co-culture spheroids, which is about 400 µm at the end of its formation (4 days) (Figure 4 and Figure 5b), does not exceed the maximum diameter of human pancreatic islets, which is 500 µm [27,28]. Spheroids with this dimension have a great advantage to be easily manipulated during media change, since they are visible to the naked eye, and to be transferred to downstream analysis without high-throughput equipment. In addition, since AECs are potentially involved in beta cell differentiation, it is fundamental to set up a model that could include a higher number of AECs in comparison to our previously developed AEC-only spheroids. This consideration was impossible at an AEC:WJ-MSC ratio of 1:2, confirming the selection of the one-to-one ratio. Moreover, in our co-culture model, AECs (Pan-Ck positive cells) localized spontaneously at the periphery of the spheroid (Figure 7), as following with increasing spheroid diameter would contain a higher number of AECs than smaller spheroids, still resembling the minimum size of human pancreatic islets [28,29] (e.g., spheroid with 50 µm diameter). We hypothesize that a greater number of AECs on the surface of the spheroid may better enable the release of insulin derived from an eventual successful differentiation into beta cells. The assembled spheroid co-cultures are in the range of measurement of an innovative instrument, the W8 Physical Cytometer, able to analyze the mass density of the spheroids with a label-free approach [30]. To further investigate the bio-physical characteristics of the assembled spheroid co-cultures, W8 analysis was performed (Figure 5). The results showed that the mono-culture control of only WJ-MSCs was quite stable after formation, but the co-culture spheroids decreased their diameter and increased their mass density over time. These results find a correlation with the immunofluorescence analysis of different ECM proteins secreted within the spheroid formation (Figure 8). Type I collagen and fibronectin belong to the interstitial matrix network that is interspersed around the cells. The deposition of fibronectin and collagen type I, usually secreted by MSCs and stromal cells [13], was visible in the spheroids at 4 days after cell seeding, the time required for consistent spheroid formation in both mono-cultures and co-cultures. The formation of the ECM network is fundamental not only for spheroid formation, but also for mirroring the micro-environment present in pancreatic islets. Indeed, laminin and type I collagen are naturally present in the pancreatic islets [23] and fibronectin is fundamental for their physiologic development, and it is thought to play a role in endocrine cell motility during islet formation [22]. Moreover, ECM can enhance secreted factor storage and differentiating agent retention; thus, the appearance of a 3D mesh of fibronectin and collagen I may support the induction protocol of differentiation [31,32].

Laminin is a protein occurring in the basal membrane, which is a lining structure of the ECM at the interface between the epithelium and the stromal compartment of several organs [20]. Laminin staining was found, only in co-culture spheroids, close to the region occupied by AECs, likely suggesting that they are the main secreting cell type, as already demonstrated in previously developed AEC-only spheroids [4]. In addition, cell–cell interaction is demonstrated with cadherin staining, a suggested marker of compaction of the spheroids [15]. By increasing inter-cellular contacts, the diameter is likely to decreased over time. However, despite the ECM still being present at 14 days of culture, the nuclei of a few cells in the core of the spheroids were lost (Figure 4 and Figure 8), where more WJ-MSCs were present in comparison to AECs edging the spheroid co-culture structure. As a consequence of this, WJ-MSCs could work better as ECM producers more than cell players during beta cell induction. Thus, the increase in cell density during long-term cultures could be a result of an augmented ratio of ECM:MSCs in the spheroid. 

An important consideration is that spheroids derived from perinatal isolated cells, unlike organoids derived from insulin-producing cells or tissues, do not possess self-renewal capacity. A prolonged time in culture can be detrimental to cell viability. For this reason, is fundamental to also consider culture duration ahead of starting the differentiation protocol. Long-term cultured spheroids suffer metabolites and gas exchange, similar to tumorspheres; the hypoxic level can increase with negative consequences and apoptotic/necrotic cores can form in the inner part of the cell aggregates. Overall, our co-culture characterization indicates short-term cultured spheroids including AECs and WJ-MSCs are superior to long-term cultured spheroids, at least for our static culture conditions. Hyperoxia, often characterizing in vitro manipulation conditions, may cause oxidative stress by the generation of reactive oxygen species that will affect the functionality of cultured stem cells. On the contrary, a controlled level of hypoxia is a physiological stimulus characterizing many stem cell niches, including the MSC one [33]. Hyperoxia can sustain stemness maintenance in vitro [34,35] and control the properties of in vitro cultured WJ-MSCs [36,37,38]. The hypoxia level did not change between 4 days and 14 days (Figure 9), but cell viability evaluated by both a metabolism indicator (Figure 3a) and a cell-membrane-integrity signal (Figure 3b) decreased. Based on our results, spheroids taken soon after their formation could be more suitable to proceed to any induction of differentiation, owing to a better viability rate.

In the future, our designed co-cultures can be optimized for dynamic culturing with bioreactors [39], in an effort to maintain the properties of the spheroid seen at its formation. 

Another limitation of a co-culture is the difficulty to dissociate the spheroids to perform subsequent analyses of the single-cell type. Preliminary experiments of dissociation through enzymatic and mechanical separation were unsuccessful. A very low yield in terms of cell number and low viability levels was recovered, in particular, after the onset of intercellular junctions at both homo- and hetero-typic levels, and following the formation of strong attachment to the ECM. Moreover, any subsequent analyses of the single-cell type should require additional protocols to isolate it, creating an issue, for example, in gene expression analysis. 

Regarding the viability of our model, based on our results (Figure 3 and Figure 4), we would likely lose WJ-MSCs if a long-term culture were to be applied. An aspect to take strongly into consideration is that such long-term cultures mirror an undifferentiated state by employing a basal cell medium. The differentiation medium may sustain stromal MSC viability or even stimulate proliferation. However, we demonstrated that AECs, which are the best candidate for becoming beta cell replacements, are still detected 14 days after seeding, suggesting that AECs can entirely survive before their induction towards endo-pancreatic differentiation. A proper differentiation into a pancreatic lineage seems to require at least more than the time used to generate our spheroid co-cultures. This is not relevant if freshly generated spheroids were to be induced. Since AEC populations present a well-known inter- and intra-donor variability, future protocols could take advantage of this and involve the use of instruments for high-throughput, gentle, label-free cell selection [30,40]. 

## 5. Conclusions

The availability of new treatments for diabetes based on immunomodulation and differentiation strategies is currently unsatisfactory as an alternative to exogenous insulin administration. By using ethical-issue-free perinatal stem cells, we developed co-culture spheroids that resembled the epithelial-stromal organization and tissue features of pancreatic islets. The characterized spheroids can be reproducibly obtained without expensive equipment; they retained cell viability and soon matured as secretors of an ECM network. During and after short-term culture spheroids can be monitored for their biophysical characteristics, thus increasing the quality and homogeneity of the co-cultures. The possibility to employ spheroids made in a few days with an easy and cost-effective method as here reported would revolutionize the diabetes stem cell therapy approach.

## Data Availability

Not applicable.

## Supplementary Materials

The following supporting information can be downloaded at: https://www.mdpi.com/article/10.3390/bioengineering10020189/s1, Figure S1: Hematopoietic markers, Video S1: IncuCyte^®^ time-lapse merge, Video S2: IncuCyte^®^ time-lapse single florescence red, Video S3: IncuCyte^®^ time-lapse single florescence green.
