# Supplementary figures and images for: Characterization of Perinatal Stem Cell Spheroids for the Development of Cell Therapy Strategy
Source: Bioengineering (Basel). 2023 Feb 2;10(2):189. doi: 10.3390/bioengineering10020189 (PMC9952228; doi:10.3390/bioengineering10020189)

a)

### AECs

CD34

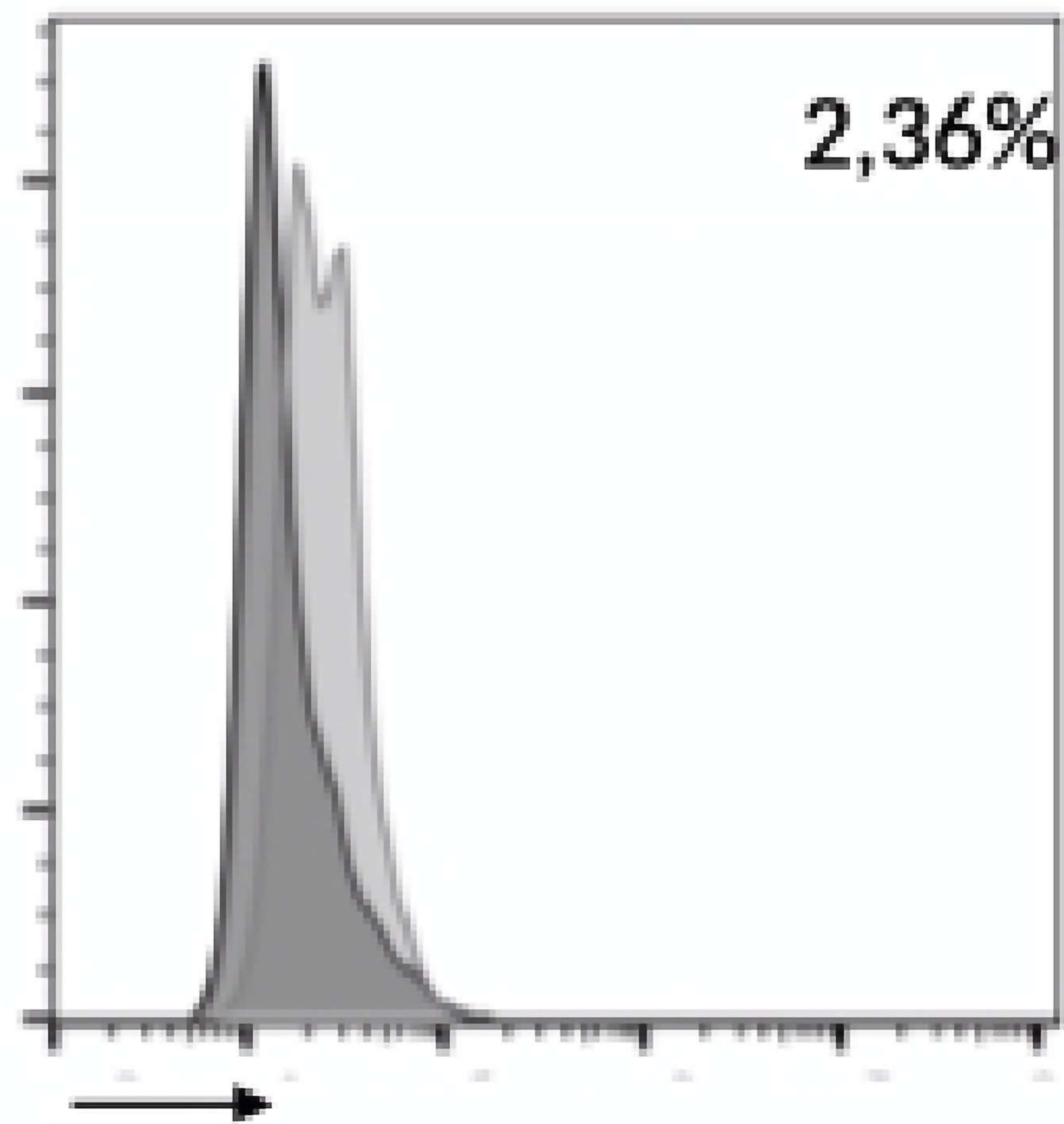

CD45

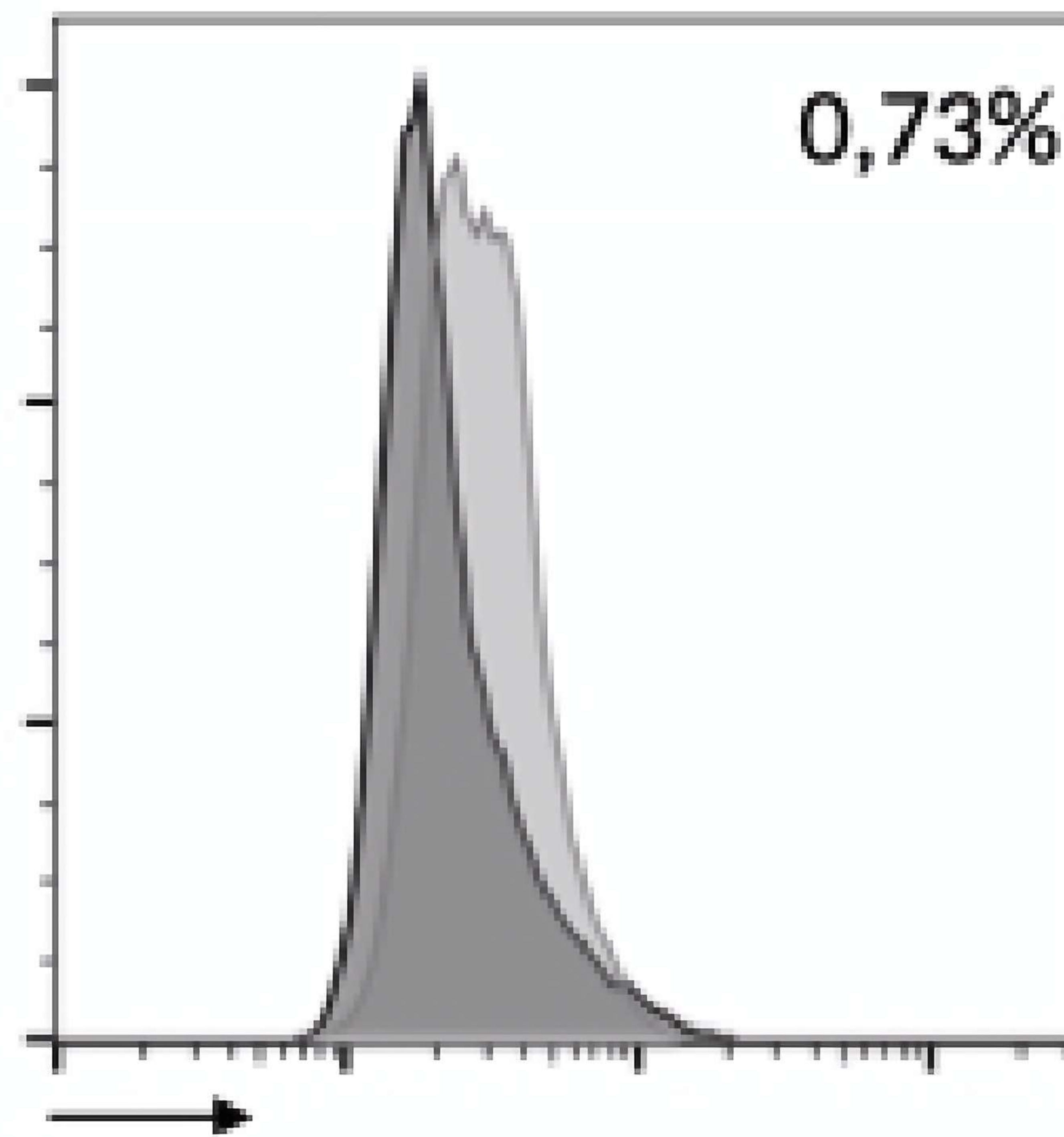

CD14

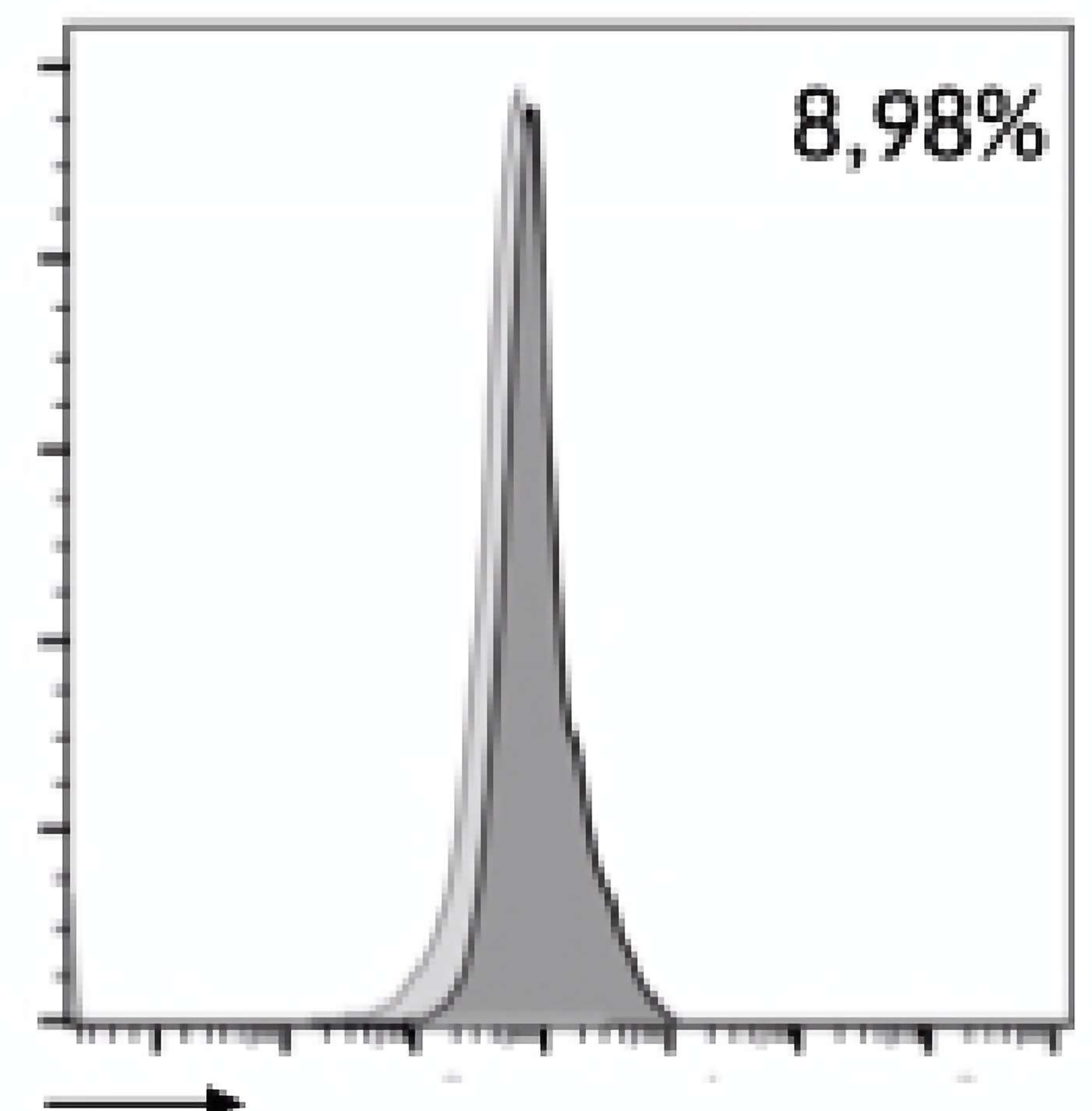

b)

### WJ-MSCs

CD34

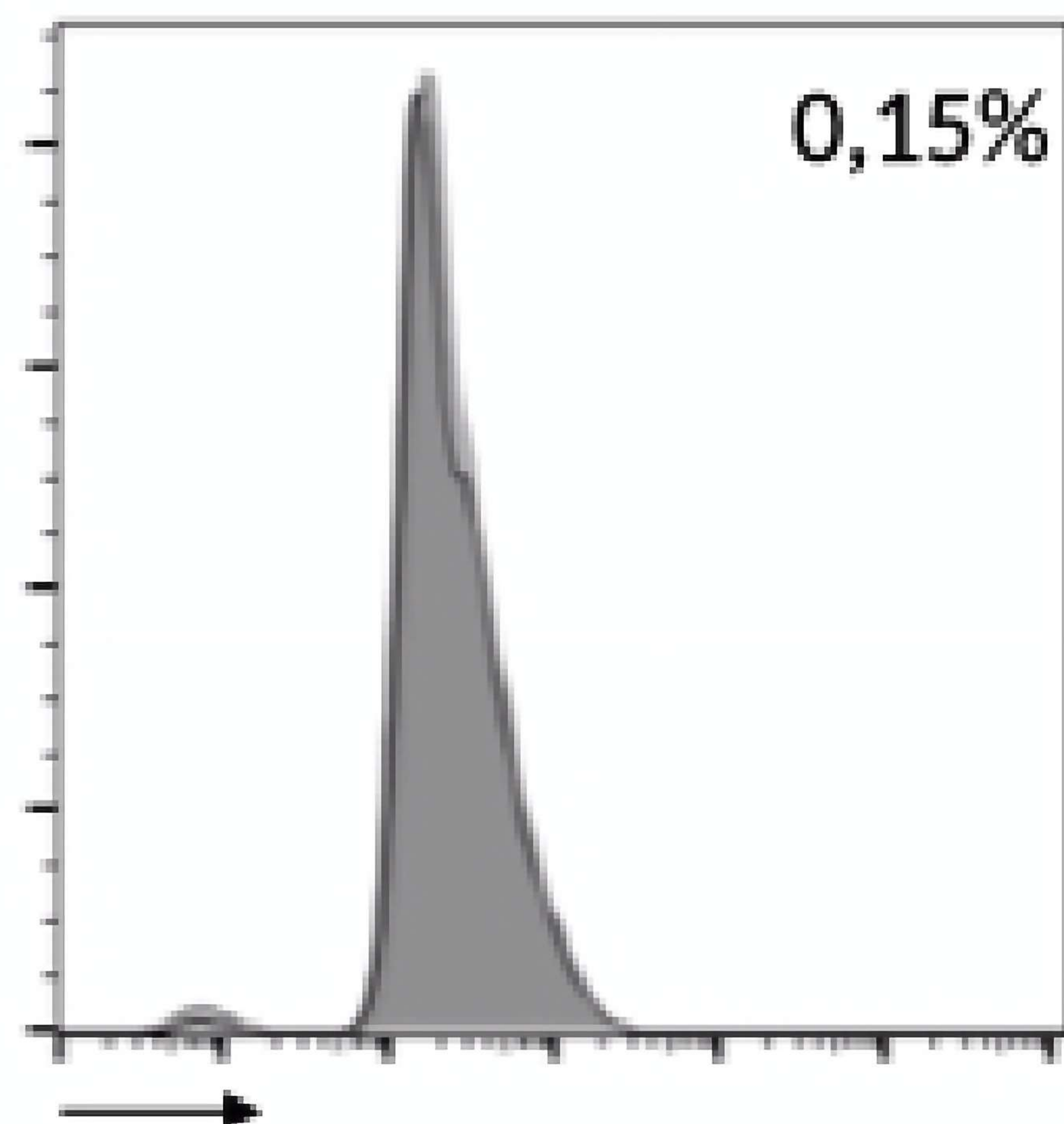

CD45

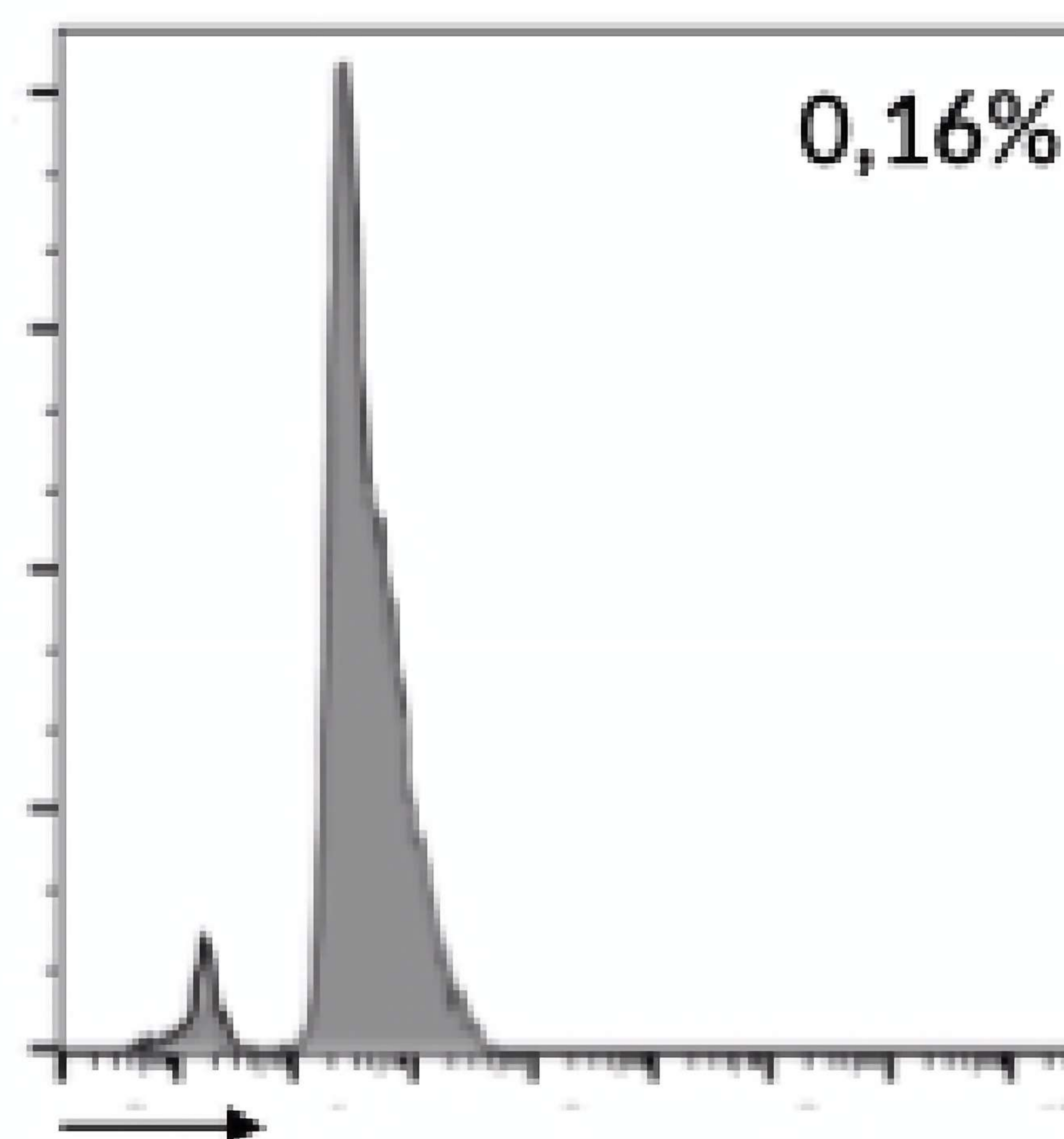

CD14

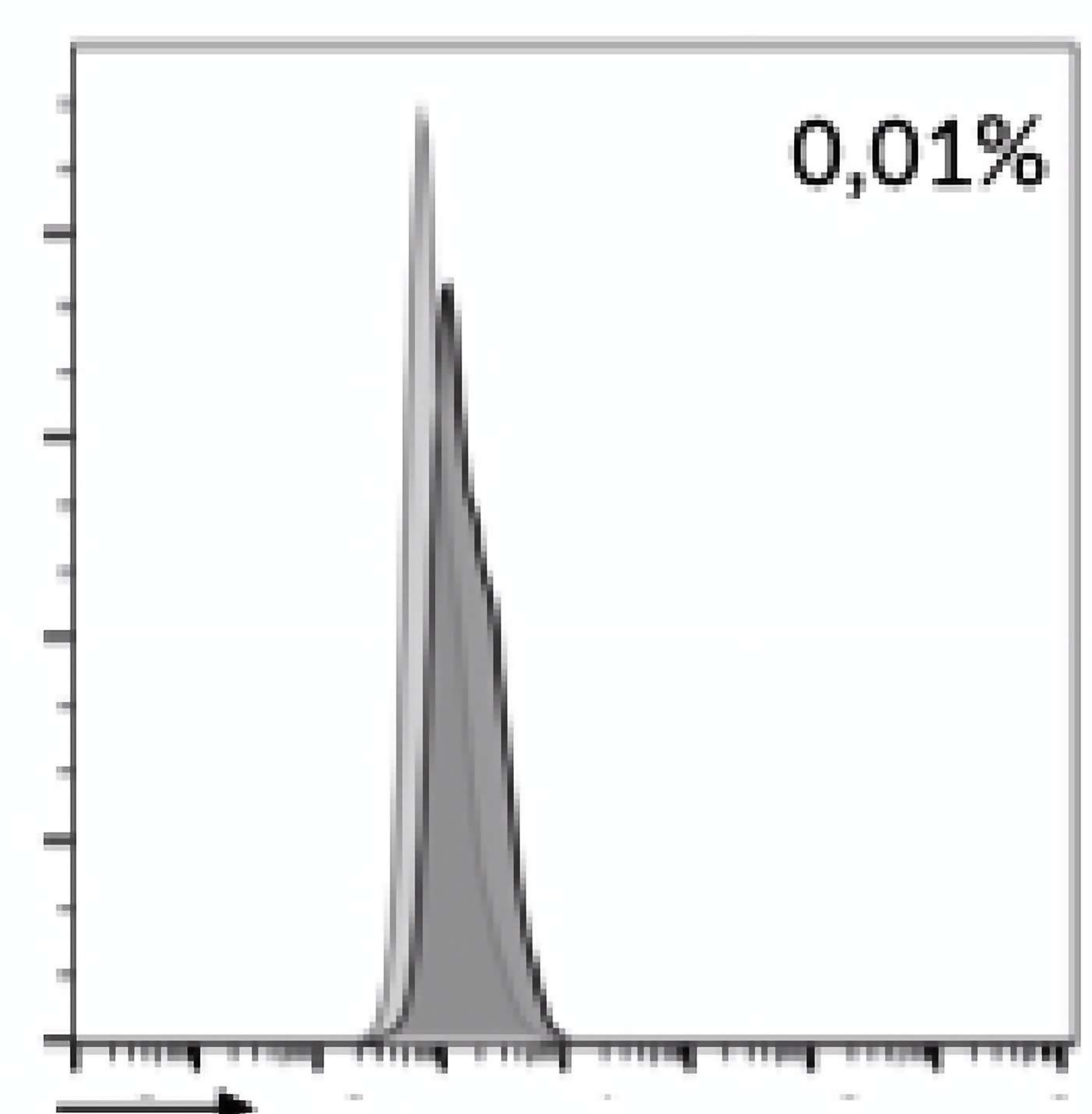

Supplement: Supplementary file 1 [file bioengineering-10-00189-s001.zip › Figure_S1.pdf]
